# *Leishmania* Parasites Differently Regulate Antioxidant Genes in Macrophages Derived From Resistant and Susceptible Mice

**DOI:** 10.3389/fcimb.2021.748738

**Published:** 2021-10-15

**Authors:** Haifa Bichiou, Sameh Rabhi, Cherif Ben Hamda, Cyrine Bouabid, Meriam Belghith, David Piquemal, Bernadette Trentin, Imen Rabhi, Lamia Guizani-Tabbane

**Affiliations:** ^1^ Laboratory of Medical Parasitology, Biotechnology and Biomolecules, Institut Pasteur de Tunis, Tunis-Belvedere, Tunisia; ^2^ Faculty of Sciences of Tunis, Université de Tunis El Manar, Tunis, Tunisia; ^3^ Department of Immunology, Institut Pasteur de Tunis, University Tunis El-Manar, Tunis, Tunisia; ^4^ Acobiom, Grabels, France; ^5^ Higher Institute of Biotechnology at Sidi-Thabet, Biotechpole Sidi-Thabet, University of Manouba, Sidi-Thabet, Tunisia

**Keywords:** *Leishmania*, macrophage, susceptibility, resistance, NRF2, antioxidants

## Abstract

Macrophage–*Leishmania* interactions are central to parasite growth and disease outcome. Macrophages have developed various strategies to fight invaders, including oxidative burst. While some microorganisms seem to survive and even thrive in an oxidative environment, others are susceptible and get killed. To counter oxidative stress, macrophages switch the expressions of cytoprotective and detoxifying enzymes, which are downstream targets of the nuclear factor erythroid 2-related factor 2 (Nrf2), to enhance cell survival. We have explored the transcription of NRF2 and of its target genes and compared the effect of the parasite on their transcription in bone marrow-derived macrophages (BMdMs) from *Leishmania*-resistant and *Leishmania*-susceptible mice. While heme oxygenase 1 (HO-1) transcription is independent of the genetic background, the transcription of glutathione reductase (Gsr) and of cysteine/glutamate exchange transporter (Slc7a11), involved in glutathione accumulation, was differentially regulated in BMdMs from both mouse strains. We also show that, except for HO-1, known to favor the survival of the parasite, the transcription of the selected genes, including Gsr, CD36, and catalase (CAT), was actively repressed, if not at all time points at least at the later ones, by the parasite, especially in Balb/c BMdMs. Consistent with these results, we found that the silencing of NRF2 in this study increases the survival and multiplication of the parasite.

## Introduction

Leishmaniasis is a parasitic disease caused by the protozoa *Leishmania*. This disease is spread worldwide and causes different clinical manifestations ranging from cutaneous lesions healing spontaneously to visceral leishmaniasis, which is the most serious form of the disease that is fatal in the absence of treatment. *Leishmania* parasites alternate between two life stages, and the flagellated promastigotes injected by the female sand fly transform into amastigotes when infecting host immune cells. As a main host for *Leishmania* replication, macrophages play a critical role in the outcome of the disease and in the battle between these two players: the host fighting the invaders with all available arsenal and the parasites developing various strategies to subvert the microbicidal functions of their host cells (Conceição-Silva and Morgado, 2019). Indeed, macrophages encounter *Leishmania* by employing an array of directly antimicrobial mechanisms such as the generation of reactive oxygen species (ROS) and reactive nitrogen species (RNS), which are highly destructive to *Leishmania*.

These oxidant compounds, while crucial in clearing invading pathogens, may cause oxidative damage to the host cells. Cells have developed different antioxidant defense systems to counter oxidative stress. These include the thioredoxin (Trx) and glutathione (GSH) systems, the two major thiol-dependent antioxidant mechanisms in cells, and heme oxygenase 1 (HO-1), which catalyzes the rate-limiting step of heme oxidation to biliverdin, carbon monoxide, and free ferrous iron (Tonelli et al., 2018). Most of these cytoprotective and detoxifying enzymes that enhance cell survival are downstream targets of the nuclear factor erythroid 2-related factor 2 (Nrf2).

Nrf2 is a stress-responsive transcription factor encoded by the *NFE2L2* gene in humans and is a member of the CNC (“cap ‘n’ collar”) subfamily of basic region leucine zipper (bZIP) transcription factors (Sykiotis and Bohmann, 2010). During homeostasis, Nrf2 is sequestered in the cytoplasm by Kelch-like ECH-associated protein 1 (Keap1, Nrf2 repressor protein), which drives NRF2 to ubiquitin-dependent proteasomal degradation (Baird and Yamamoto, 2020). After redox perturbation, Nrf2 is released from Keap1 and translocates into the nucleus. It then forms heterodimers with small Maf proteins and binds antioxidant response elements (AREs) regulating a broad range of expressions of antioxidant and detoxification genes (Itoh et al., 1999). The promoter of the Nrf2 gene itself contains AREs and amplifies the redox cascades *via* positive feedback regulation (Nguyen et al., 2003).

To clarify the contribution of the Nrf2/HO-1 axis and that of other detoxifying enzymes during *Leishmania major* infection, we explored the activation of Nrf2 and the transcription of target genes in murine macrophages from mice resistant or susceptible to *Leishmania* parasite infection.

## Materials and Methods

### Ethics Statement

All mouse work was done according to the directive 86/609/EEC of the European Parliament and of the Council on the Protection of Animals Used for Scientific Purposes. Approval for mouse experiments was obtained from the Ethics Committee of Institute Pasteur of Tunis, with ethics approval no. 1204.

### Parasites and Cell Culture

*L. major* (MHOM/TN/95/GLC94 zymodeme MON25) were maintained at 26°C in RPMI 1640 medium (Sigma, Taufkirchen, Germany) supplemented with 10% heat-inactivated fetal calf serum (Gibco, Waltham, MA, USA), penicillin (200 U/ml), streptomycin (200 µg/ml), and glutamine (4 mM). In all assays, promastigotes in the stationary phase of growth (5–6 days) were washed in culture medium at 3,000 rpm for 10 min at 22°C, resuspended in medium again, and used for infection. Parasites are killed by a 10-min incubation at 100°C. Ds-Red promastigotes were generated as previously described (Rabhi et al., 2016). Parasites were maintained in culture in medium containing 25 μg/ml hygromycin B.

The mouse macrophage leukemia cell line Raw264.7 (ATCC, Manassas, VA, USA) was maintained in RPMI 1640 medium (Sigma) supplemented with 10% fetal bovine serum (FBS; Gibco), penicillin (200 U/ml), streptomycin (200 µg/ml), and glutamine (4 mM; SigmaG1146) at 37°C in a humidified incubator with 5% CO_2_. Macrophages were cultured in 6-well plates and incubated for 12–24 h at 37°C with 5% CO_2_ to adhere and then infected with *Leishmania* promastigotes at a ratio of 10:1. Macrophages interacting with *L. major* were maintained at 37°C with 5% CO_2_. The infected cells were then harvested at different time points. Infections were confirmed using Giemsa staining.

Balb/c and C57BL/6 mice (Elevage Janvier, Le Genest-Saint-Isle, France) were killed and their hind legs removed for the isolation of bone marrow-derived macrophages (BMdMs). Briefly, the femurs and tibias were flushed with RPMI 1640 using a 25-gauge needle. Contaminating erythrocytes were lysed through the addition of a lysis solution. All cells were incubated in T75 culture flasks at 1.5 × 10^6^ cells/ml in complete media added with 80 ng/ml macrophage colony-stimulating factor (M-CSF; Peprotech, Neuilly sur Seine, France) overnight for stromal cell elimination. Non-adherent, immature macrophages were transferred to fresh culture-treated Petri dishes (Nunc, Rochester, NY, USA) and grown for 7 days, with re-feeding on day 3 to induce macrophage differentiation. The purity of BMdMs was analyzed through the evaluation of the phenotypic expression of a specific macrophage subset surface marker (F4/80) by flow cytometry. Of the generated macrophages, 80%–90% were F4/80-positive.

### Knockdown Macrophage Generation

Raw264.7 cells were seeded 18–20 h pre-transfection in 24-well plates at a confluency of 70%. The cells were transfected using the ESCORTTM II transfection reagent (Sigma) with the empty plasmid non-target pLKO (NT-pLKO) or one of the five different NRF2 DNAs and extracted using Maxiprep from the *NFE2L2* Mission shRNA Glycerol Stock (Sigma) according to the manufacturer’s instructions.

Briefly, 2 µg of DNA was diluted and incubated for 20 min with the diluted ESCORT II reagent at room temperature to form the ESCORT II/DNA complexes. These complexes were then added to the cells growing in serum-containing culture medium and incubated at 37°C. At 24 h post-transfection, fresh growth medium was added. For selection, puromycin was added to the fresh medium 72 h post-transfection. The cells were kept in culture for 1 month with a cell medium change approximately two times a week. The cells were then cryopreserved after assessment of the silencing efficiency by Western blotting. Wild-type (WT), NT-pLKO, and si-Nrf2 cells were stimulated with 100 ng/ml lipopolysaccharide (LPS) and the intensities of the bands were compared. Cells transfected with the NRF2 clone, which gave the higher silencing percentage, and the corresponding ones transfected with the NT-pLKO plasmids were used for subsequent experiments.

### Ds-Red *Leishmania* Parasite Quantification by Flow Cytometry

We used Ds-Red-GLC94 *L. major* parasites (Rabhi et al., 2016) to infect transfected si-NRF2 and NT-pLKO cells. Seventy-two hours post-infection, the cells were immediately analyzed without fixation for the estimation of parasite multiplication by flow cytometry using a Becton Dickinson FACSCanto II flow cytometer. The analysis was fulfilled with the BD FACSDiva 6 software (Becton Dickinson, Franklin Lakes, NJ, USA).

### Protein Extraction and Cell Fractionation

Cells were harvested at different time points, washed gently with ice-cold phosphate-buffered saline (PBS), scraped, and centrifuged at 1,200 rpm for 10 min. Nuclear and cytoplasmic protein extracts were prepared using a hypotonic hypertonic buffer. The pellets were resuspended in 80 μl volume of hypotonic buffer containing 10 mM HEPES–KOH (pH 7.6), 60 mM KCl, 1 mM EDTA, 1µg/ml aprotonin, 1 mM orthovanadate, 1 mM dithiothreitol (DTT), and 1 mM phenylmethylsulfonyl fluoride (PMSF) for isolation of the cytoplasmic extract. After incubation on ice for 10 min, 1/30 on the volume of 10% NP40 was added for 1 min and the samples then centrifuged at 10,000 rpm for 5 min. The cytoplasmic extract was collected and stored. The nuclear pellet was resuspended in hypertonic buffer containing 10 mM HEPES–KOH (pH 7.6), 1.5 mM MgCl_2_, 420 mM NaCl, 0.2 mM EDTA, 25% glycerol, 1µg/ml aprotonin, 1 mM orthovanadate, 1 mM DTT, and 1 mM PMSF. The nuclear pellets in hypotonic buffer were vortexed to homogenize the lysate, sonicated using Vibra-Cell at amplitude of 60% for three cycles of 10 s with 10-s pauses while being kept, and incubated on ice for 30 min. The lysates were then centrifuged at 14,000 rpm for 20 min to pellet any cell debris and the supernatants containing the nuclear proteins were collected into new tubes. For whole cell lysis, we used 30 µl of lysis buffer containing 10 mM Tris–HCl (pH 7.5), 50 mM NaCl, 50 mM sodium fluoride (NaF), 2 mM EDTA, 1 mM EGTA, 2% Nonidet P-40 (NP-40), 0.75% sodium deoxycholate (DOC), 1 mM orthovanadate, 1 µg/ml aprotinin, 1 mM PMSF, and 1 mM DTT. After 10 min incubation on ice, the extracts were centrifuged at 15,000 rpm for 20 min. All extracts were stored at −20°C. The protein content of each compartment was determined using the bicinchoninic acid (BCA) protein assay (Sigma).

### Western Blotting

Equal amounts of protein were separated by 10% sodium dodecyl sulfate polyacrylamide gel electrophoresis and transferred onto a 0.45-µm polyvinylidene difluoride (PVDF) immunoblot membrane (Amersham, Amersham, UK). After membrane blocking with 5% skimmed milk in PBS with 0.01% Tween 20 (wash buffer) for 1 h at room temperature (RT), the membranes were incubated overnight at 4°C with commercial primary antibodies against phospho-AKT (Cell Signaling, Danvers, MA, USA), HO-1 (Santa Cruz Biotechnology, Dallas, TX, USA), NRF2 (Santa Cruz Biotechnology), histone (Cell Signaling), β-actin (Sigma-Aldrich, St Louis, MO, USA), and PARP (Cell Signaling). The next day, the membranes were washed three times and then incubated with horseradish peroxidase-conjugated anti-rabbit secondary antibody (1:2,000; Dako, Carpinteria, CA, USA) for another 1 h at RT. Blots were visualized with a densitometric ECL kit (Amersham) and analysis was performed using ImageJ software.

To analyze the effects of kinase inhibitors on the expressions of Nrf2 proteins, seeded raw cells in dishes were pretreated with wortmannin (200 mM) for 3 h; then, the cells were washed and infected with the *Leishmania* promastigotes for different times. The cell lysates prepared using the lysis buffer, as described above, were subjected to Western blot analysis.

### RNA Extraction and qRT-PCR

Total RNA from macrophages was isolated using the TRIzol reagent (Sigma). Total RNAs from uninfected and infected macrophages were prepared using the RNeasy mini kit (Qiagen, Hilden, Germany). RNA quantity was controlled using the NanoDrop ND-1000 micro-spectrophotometer, and RNA quality and integrity (RNA integrity no. RIN.9) were monitored on the Agilent RNA Pico LabChips (Agilent Technologies, Palo Alto, CA, USA). Reverse transcriptions were performed for each sample in a final reaction volume of 20 µl with 273 ng of total RNA using 200 U of SuperScript III enzyme (M-MLV Reverse Transcriptase, Invitrogen) and 250 ng of random primers according to the manufacturer’s instructions (25°C for 10 min, 42°C for 50 min, and 70°C for 15 min). A negative control was included by performing reverse transcription with no template. Quantitative PCR (qPCR) experiments were carried out using EVA Green chemistry on a BioMark qPCR apparatus (Fluidigm, San Francisco, CA, USA) following the manufacturer’s instructions. For each cDNA sample, specific target amplification (STA) was performed with a pool of primers targeting all selected genes (pre-amplification of 14 cycles using the TaqMan PreAmp Master Mix, Applied Biosystems, Waltham, MA, USA) and following the manufacturer’s instructions. Each qPCR was performed with 1/20 STA dilution, in duplicate. Relative gene expression kinetics was created by a first normalization with four reference genes followed by a second normalization with non-infected (NI) macrophage cells. Values are expressed in fold changes (2^−ΔΔ^ *^CT^* method) compared to NI macrophage cells.

### Statistical Analysis

Data were presented as the mean and SD (standard deviation). All graphs generated and the related statistical analysis were performed using GraphPad Prism. Statistical analysis was performed using ANOVA. Significance was reached with *p*-values <0.05. *P*-values were shown as *p < 0.05, **p < 0.01, ***p < 0.001, and ****p < 0.0001.

## Results

### *Leishmania* Infection Drives HO-1 Expression

**Figure 1:**
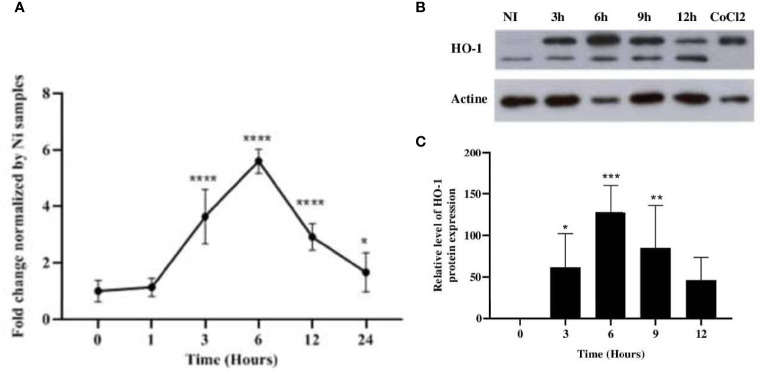
*Leishmania major* induced the expression of HO-1 at both the mRNA and protein levels. Balb/c bone marrow-derived macrophages (BMdMs) and/or Raw264.7 cells were infected for the indicated time periods with *L. major* promastigotes. **(A)** The extracted mRNAs were used to perform RT-PCR. **(B)** The expression of heme oxygenase 1 (HO-1) was evaluated at the protein level by immunoblotting. CoCl_2_ stimulation (250 ng/ml) for 3h was used as control. **(C)** Densitometric quantification of HO-1 protein levels using ImageJ. All data are expressed as the mean ± SD from three independent experiments. Statistical significance was placed at *p* < 0.05. *p < 0.05, **p < 0.01, ***p < 0.001, ****p < 0.0001 *vs*. non-stimulated cells (two-way ANOVA).

We first assessed the ability of *L. major* parasites to induce the expression of HO-1. Balb/c BMdMs and/or Raw264.7 macrophages were infected with *L. major* promastigotes. The infected cells were harvested and the expressions of HO-1 messenger RNA (mRNA) and proteins were analyzed. Our results showed that promastigotes significantly induced the transcription and the protein expression of HO-1 (**Figure 1**).

### *L. major* Infection Leads to Activation of the Nrf2 Pathway

Because the expressions of antioxidant genes are under the control of the Nrf2 transcription factor (TF), the activation of this master TF was investigated.

**Figure 2:**
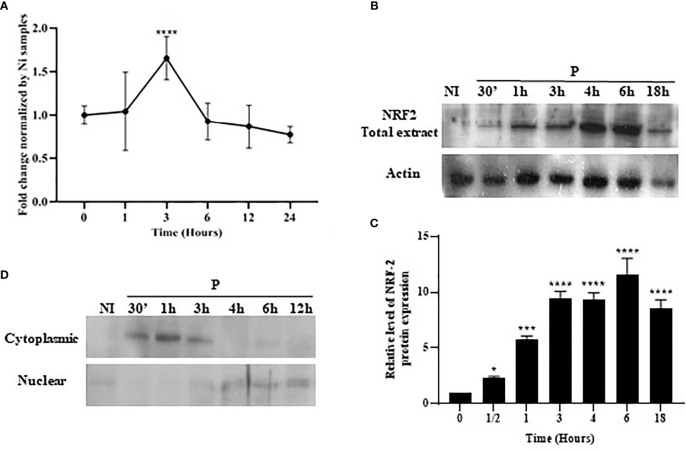
Stimulation and nuclear accumulation of Nrf2 in response to *Leishmania major* infection. Balb/c bone marrow-derived macrophages (BMdMs) and/or Raw264.7 cells were infected for the indicated time period with *L. major* promastigotes. **(A)** The extracted mRNAs were used to perform RT-PCR. **(B)** Total Nrf2 expression was evaluated at the protein level by immunoblotting. **(C)** Densitometric quantification of the NRF2 protein levels using ImageJ. All data are expressed as the mean ± SD from two independent experiments. *p < 0.05, ***p < 0.001, ****p < 0.0001 *vs*. non-stimulated BMdMs (two-way ANOVA). **(D)** Total protein extracts were fractionated into cytoplasmic and nuclear extracts and immunoblots performed with an anti-Nrf2 antibody. ERK1/2 and PARP were used to confirm the efficacy of cytosol and nuclear fractionation.

BMdMs from Balb/c mice or Raw264.7 cells were infected with *L. major* promastigotes for different times. The mRNA and protein were extracted for RT-PCR and Western blotting. The quantitative RT-PCR (qRT-PCR) results showed a slight but significant increase in the expression of Nrf2 mRNA, which peaked at 3 h post-infection (hpi) (**Figure 2A**). The parasite-induced expression of the NRF2 protein was detected as early as 30 min pi, and the abundance peaked between 4 and 6 hpi before declining progressively (**Figure 2B**). These data suggested an upregulation of the expression of Nrf2 by the increased transcription of the Nrf2 gene leading to increased Nrf2 protein levels in infected cells. To confirm the activation of the Nrf2 pathway, we visualized the nuclear translocation of the Nrf2 protein. Subcellular fractionation showed Nrf2 accumulation in the nucleus following infection. Nrf2 was, during the first hour post-infection, predominantly localized in the cytoplasm. At 3 hpi, the Nrf2 protein started to be exported to the nucleus, and the nuclear localization of NRF2 was observed until 12 hpi (**Figure 2C**).

### PI3K/Akt and NRF2 Regulate the Expression of HO-1

**Figure 3:**
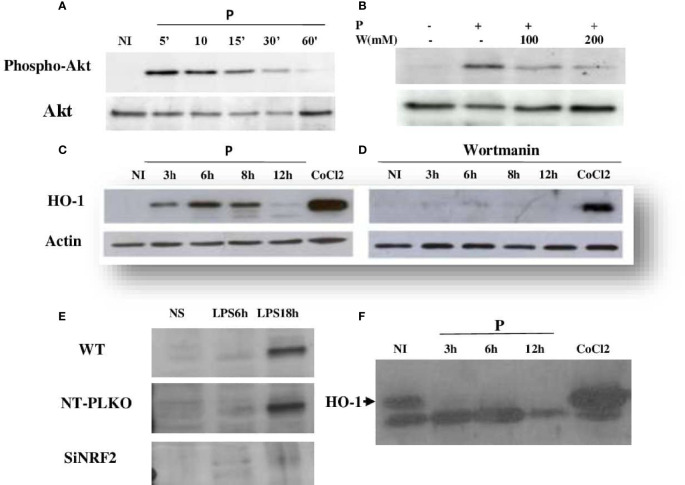
*Leishmania major* induced HO-1 expression through PI3K/Akt and the Nrf2 transcription factor. **(A, C)** Raw264.7 cells were infected for the indicated time periods with *L. major* promastigotes and the expressions of Akt and HO-1 assessed using Western blot. CoCl_2_ stimulation for 3 h (250 ng/ml) was used as the control. **(B, D)** Macrophages were pre-incubated with wortmannin (200 mM) for 3 h, followed by infection with *L. major* promastigotes for different times. Akt phosphorylation and HO-1 expression were determined by immunoblotting. **(E)** Raw264.7 cells were transfected (24 h) with either Nrf2 (Si) or control siRNA (NT-pLKO), followed by infection with lipopolysaccharide (LPS) for 18 h, lysed, and examined by Western blot for Nrf2 expression. **(F)** *NRF2* ^−/−^ cells were infected for different times with *L*. *major* promastigotes, lysed, and examined by Western blot for HO-1 expression.

To determine the upstream regulators of HO-1, macrophages were infected or pretreated with a phosphatidylinositol 3-kinase (PI3K) inhibitor, wortmannin, and then infected by *L. major* promastigotes. Our results showed that wortmannin treatment decreased the parasite-induced phosphorylation of Akt (**Figures 3A, C**) and the expression of HO-1 protein (**Figures 3B, D**), showing that PI3K/Akt activity is required to induce HO-1 expression during *Leishmania* infection.

To investigate the antioxidant function of Nrf2, Raw246.7 si-NRF2 knockdown cells were generated. As expected, cells with disrupted Nrf2 did not express the silenced TF in response to LPS stimulation (**Figure 3E**). The effect of Nrf2 knockdown on the immunomodulatory expression of HO-1 protein was investigated with Western blot. As shown in **Figure 3F**, the silencing of Nrf2 strongly reduced the expression of HO-1 in response to *Leishmania* infection, suggesting that, in response to *Leishmania* infection, the increased expression of HO-1 is dependent on the Nrf2 TF.

### Activation of NRF2 and HO-1 Transcription by *Leishmania* Parasites Is Independent of the Genetic Background

To determine whether *Leishmania* infection differentially regulates the mRNA expression of NRF2 and HO-1 in resistant and susceptible mice, Balb/c and C57Bl/6 BMdMs were infected with *L. major* promastigotes for different times. Infected cells were harvested and the expressions of antioxidant genes were analyzed using RT-PCR.

**Figure 4:**
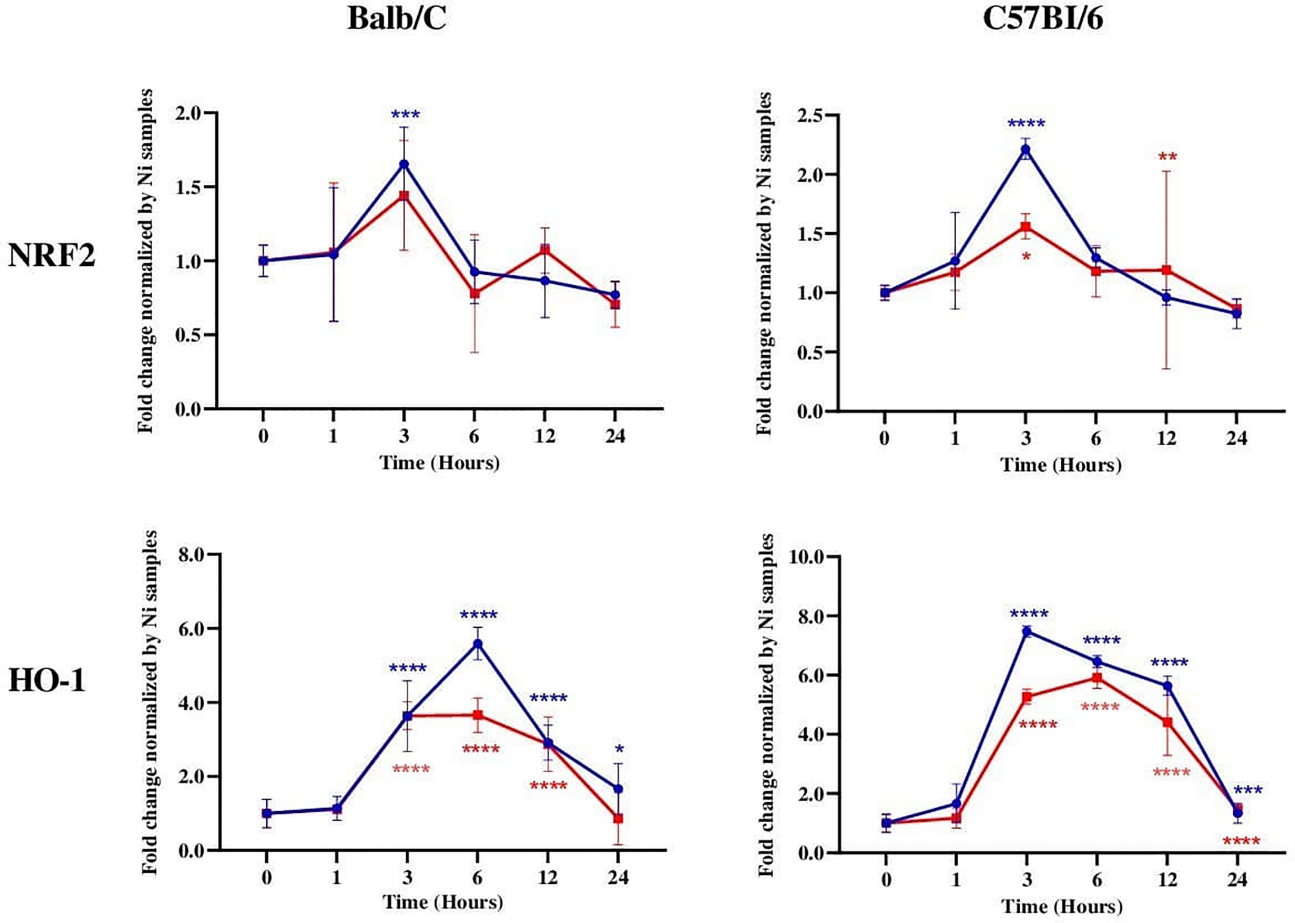
NRF2 and HO-1 mRNAs were induced in *Leishmania*-susceptible and *Leishmania*-resistant bone marrow-derived macrophages (BMdMs). Balb/c and C57Bl6 BMdMs were infected by live parasites (*P*) (*blue filled circle*) or heat-inactivated (*Kp*) (*red filled square*) *L. major* promastigotes for different times. RT-PCR targeting Nrf2 and HO-1 was performed. The graphs show fold change results expressed as the mean ± SD from three independent experiments. *p < 0.05, **p < 0.01, ***p < 0.001, ****p < 0.0001 *vs*. non-stimulated BMdMs (two-way ANOVA).

Our results showed that both Nrf2 and HO-1 mRNAs were significantly induced by the parasites in C57Bl/6 BMdMs (**Figure 4**). The transcriptional kinetics of Nrf2 is very similar in BMdMs from both mouse strains, with slightly higher expression levels in C57Bl/6 BMdMs. Regarding HO-1, the transcription peaked earlier (3 hpi), lasted longer, and was at 12 h twice as important in C57Bl/6 BMdMs. Killed parasites induced NRF2 and HO-1 to nearly the same extent as did the live parasites in BMdMs from both mouse strains.

### The Impact of *Leishmania* Infection on the Expressions of Several NRF2 Antioxidant Genes

**Figure 5:**
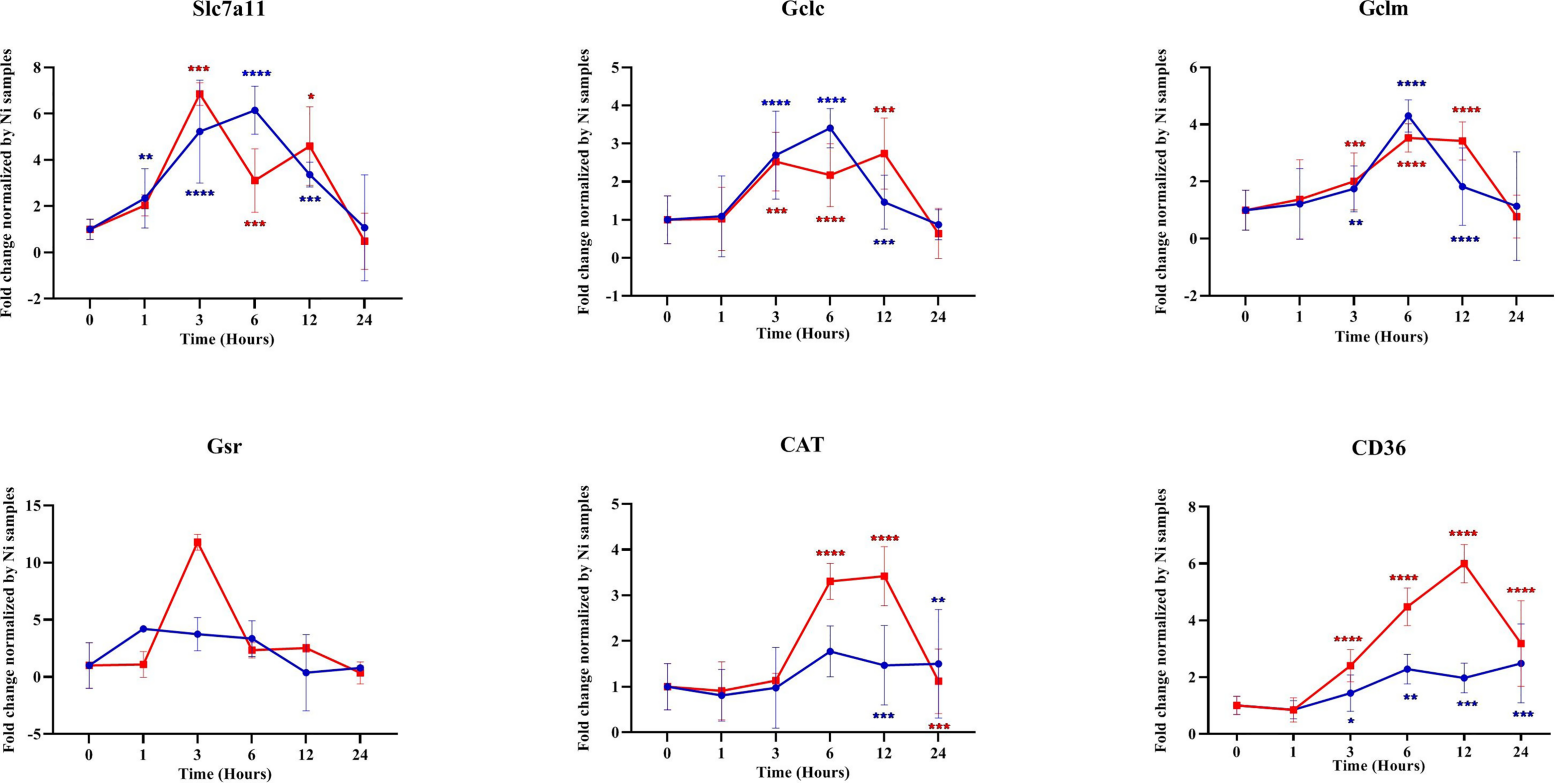
Transcription of different antioxidant genes was repressed by parasites. Balb/c bone marrow-derived macrophages (BMdMs) were infected with live parasites (*P*) (*blue filled circle* or heat-inactivated (*Kp*) (*red filled square*) *L. major* promastigotes for different times. RT-PCR targeting Slc7a11, GCLc, GCLm, Gsr, CAT, and CD36 was performed. The graphs show fold change results expressed as the mean ± SD from three independent experiments. *p < 0.05, **p < 0.01, ***p < 0.001, ****p < 0.0001 *vs*. non-stimulated BMdMs (two-way ANOVA).

**Figure 6:**
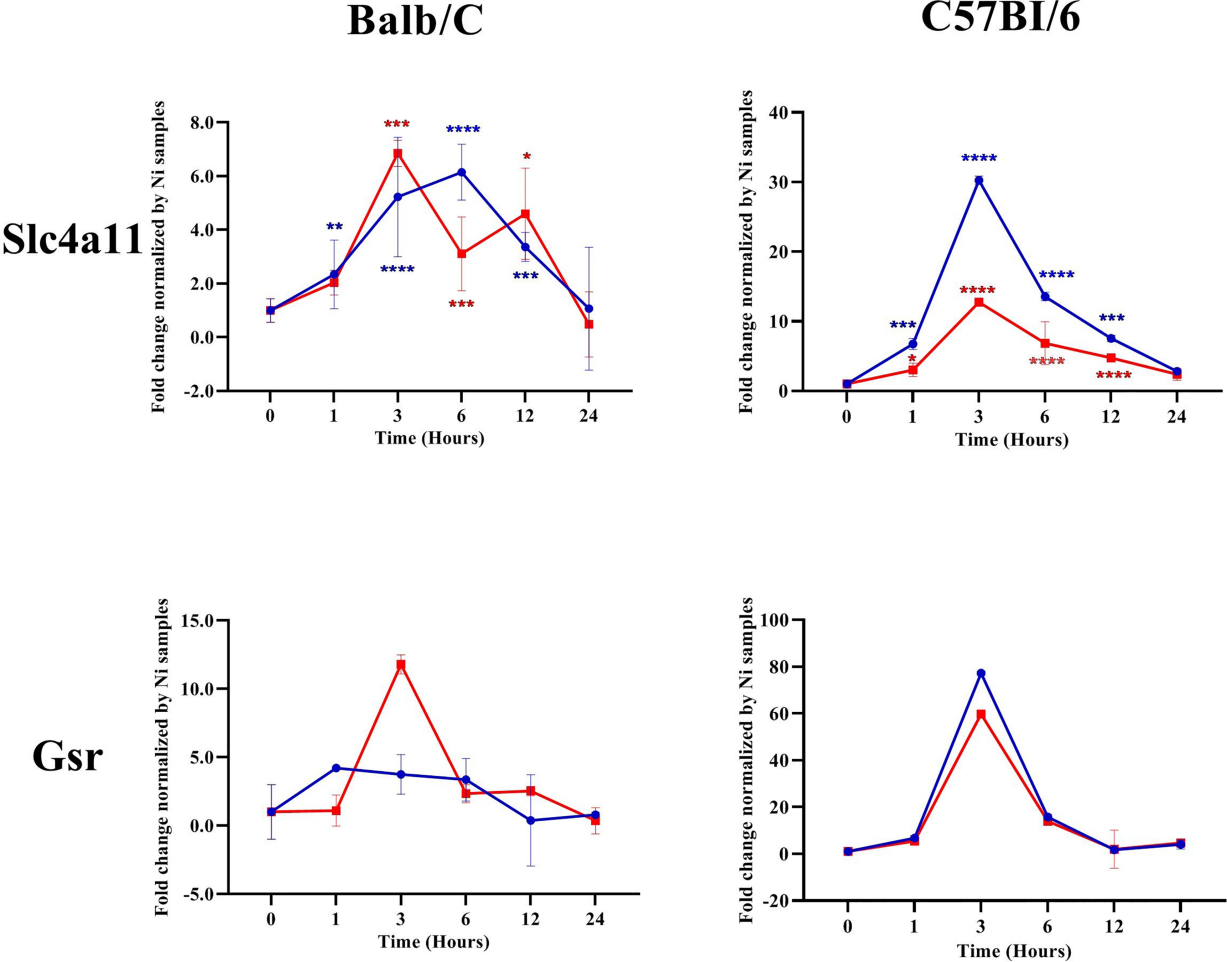
Slc7a11 and Gsr were differentially regulated by *Leishmania* parasites in Balb/c and C57Bl6 bone marrow-derived macrophages (BMdMs). Balb/c and C57Bl6 BMdMs were infected by live parasites (*P*) (*blue filled circle*) or heat-inactivated (*Kp*) (*red filled square*) *L. major* promastigotes for different times. RT-PCR targeting Slc7a11 and Gsr was performed. The graphs show fold change results expressed as the mean ± SD from three independent experiments. *p < 0.05, **p < 0.01, ***p < 0.001, ****p < 0.0001 *vs*. non-stimulated BMdMs (two-way ANOVA).

We investigated the transcription of different NRF2-regulated antioxidant genes in the infected macrophages. Our results showed that, in Balb/c BMdMs, *Leishmania* increased the transcription of the cysteine/glutamate exchange transporter (Slc7a11) regulating the cysteine influx and that of the catalytic (GCLc) and modifier (GCLm) subunits of the γ-glutamyl-cysteine ligase, the rate-limiting enzyme regulating the synthesis of glutathione (**Figure 5**). The parasites also increased the transcription of glutathione reductase (Gsr), CD36, and catalase (CAT). The transcription of almost all of the antioxidant genes peaked at 6 hpi, except for that of Gsr, which peaked earlier (3 hpi). Similar results were obtained in C57Bl/6 BMdMs, except for Slc7a11 and Gsr (**Figure 6**). Indeed, Slc7a11 mRNA was fivefold and Gsr mRNA was 20-fold higher in C57Bl/6 BMdMs. Moreover, when compared to that induced by inactivated parasites, Gsr was not only transcribed to a lesser extent but was also repressed at all time points by live parasites in Balb/c-derived macrophages. In fact, the transcription of almost all of the selected genes was actively repressed by the live parasite; this repression was observed at all time points for Gsr, CD36, and CAT and at the later times for the other genes.

### NRF2 Silencing Promotes *Leishmania* Persistence and Proliferation Inside Macrophages

**Figure 7:**
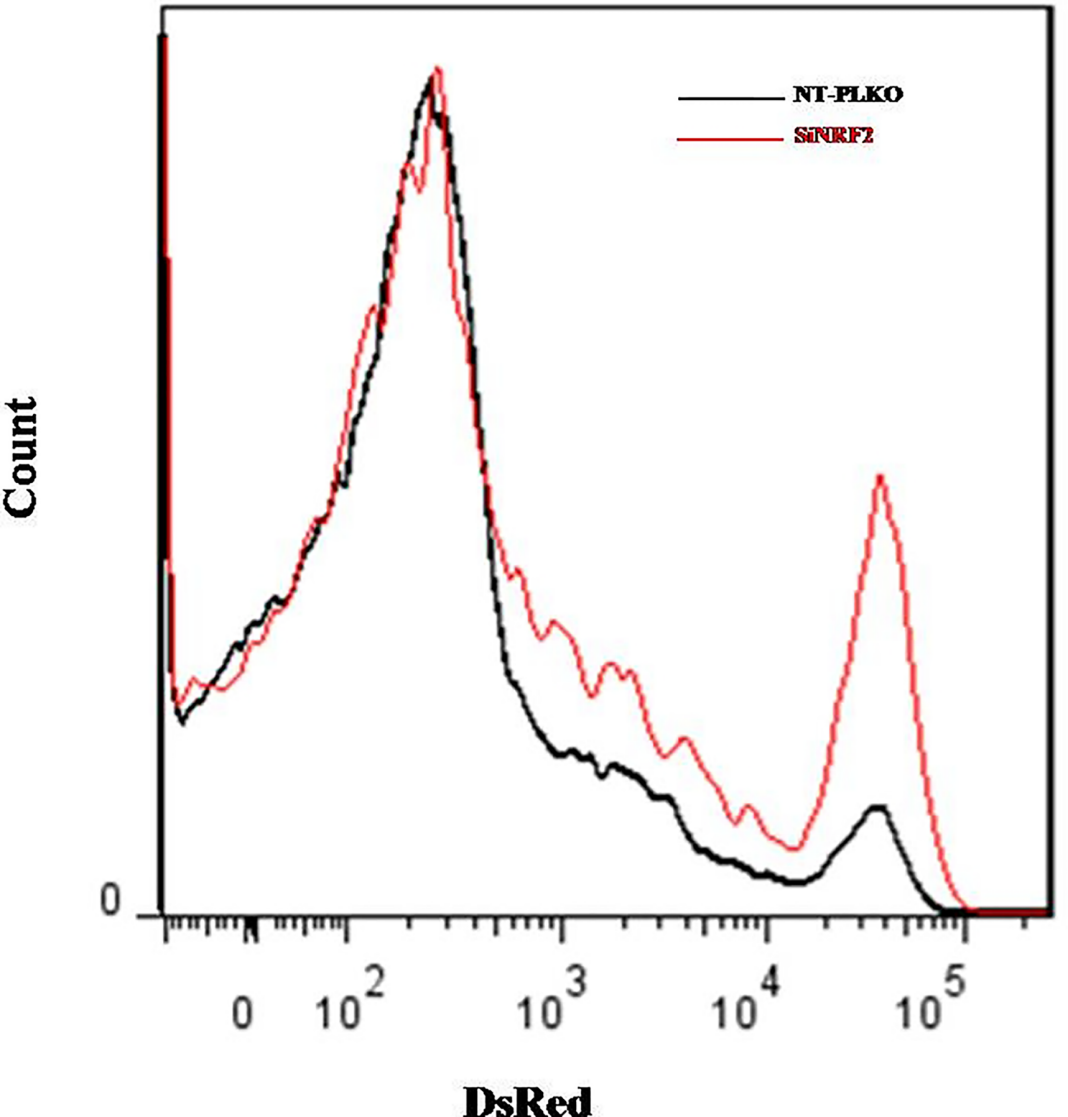
Silencing of NRF2 promoted parasite survival. NT-pLKO or si-NRF2 Raw264.7 cells were infected with Ds-Red promastigotes. Macrophages (MΦs) parasitized with Ds-Red parasites were analyzed 72 h post-infection by flow cytometry. The percentages of Ds-Red fluorescence corresponding to the multiplication of amastigotes in parasitized MΦs were compared in NT-pLKO and si-NRF2-infected macrophages.

Intracellular pathogens such as *Leishmania* use macrophages as a reservoir for dissemination. To test the ability of Nrf2 expression in controlling parasite load and multiplication, Ds-Red promastigotes were used to investigate the intracellular load of *Leishmania*. NT-pLKO raw cells and deficient NRF2 cells were infected with Ds-Red promastigotes for 72 h and the parasite load was analyzed by flow cytometry. Our experiments showed that Nrf2 loss in infected macrophages increased the ability of amastigotes to multiply inside the macrophages; parasite fluorescence emission is significantly more important in NRF2 knockdown cells (**Figure 7**).

## Discussion

Macrophage–*Leishmania* interaction and the response of macrophages to infection direct the outcome of the disease. Parasite infection activates microbicidal mechanisms such as oxidative response that help protect the host against the invaders, but can be harmful for the cells. The Nrf2 signaling pathway leads to the activation of all defense genes that aim to protect the cells from the oxidative response switched to face the infection. The Nrf2 TF plays a key role in the maintenance of intracellular redox homeostasis and the regulation of inflammation. Among the antioxidative stress genes whose expressions are regulated by NRF2, HO-1, encoded by the *Hmox1* gene, has received considerable attention. HO-1 is responsible for heme detoxification and, apart from ROS neutralization, has a potent anti-inflammatory effect and plays a critical role in iron homeostasis (Vijayan et al., 2018). The inducible HO-1 isoform is induced in response to *Helicobacter pylori* infection (Gobert et al., 2014), required for protection against *Toxoplasma gondii* (Araujo et al., 2013), and critically contributes to host resistance to *Mycobacterium* infections in mice (Silva-Gomes et al., 2013). It is also activated in response to protozoan parasites, such as *Trypanosoma brucei* (Campbell et al., 2019) and *Trypanosoma cruzi* (Paiva et al., 2012). Our results indicated that *L. major* also increased the expression of HO-1 both at the mRNA and protein levels (**Figure 1**). This result is consistent with previous studies showing an increased expression of HO-1 in macrophages in response to different species of *Leishmania* in either of its two forms (Pham et al., 2005; Luz et al., 2012; de Menezes et al., 2019; Saha et al., 2019).

HO-1 activation is dependent on the PI3K/Akt and Nrf2 signaling pathways. Indeed, we showed that *L. major*, as previously reported, induced the PI3K/Akt pathway (Ruhland et al., 2007) and the activation of the Nrf2 TF and its translocation to the nucleus (de Menezes et al., 2019). Wortmannin treatment and NRF2 silencing showed that these two pathways were both involved in the regulation of HO-1 expression (**Figure 2**).

HO-1 expression orchestrated by the activation of the Nrf2 promoter has been associated with parasite survival and persistence, as the BMdMs from Hmox1^−/−^ Balb/c mice presented a significantly reduced *Leishmania chagasi* parasite burden (Luz et al., 2012). In contrast, for other different pathogens including Trypanosomatidae, HO-1 seems to protect the host against the invaders. Indeed, inhibition of the activity of HO-1 increased *T. cruzi* parasitemia (Paiva et al., 2012). Similarly, the silencing of HO-1 in *H. pylori*-infected macrophages downregulated M1 polarization and favored *H. pylori* survival (Gobert et al., 2014). HO-1-deficient mice developed higher pathogen loads and were more susceptible to intravenous *Mycobacterium avium* infection, suggesting that HO-1 expression in macrophages is strictly required for protection against mycobacterial infection in mice (Silva-Gomes et al., 2013).

The host genetic background plays a key role in the development of leishmaniasis and has a strong impact on the severity and the final outcome of the disease. C57BL/6 and BALB/c mice, the prototypical Th1- and Th2-type mouse strains, are respectively resistant and susceptible to *Leishmania* parasites. The innate immune response of macrophages, different between these mouse strains, certainly affects the development of the adaptive immunity of Th1 and Th2 (Watanabe et al., 2004). We tried first to determine whether the expressions of NRF2 and HO-1 are different in these two mouse strains showing contrasting behaviors in response to *Leishmania* infection. Our results revealed that the transcriptions of NRF2 and HO-1 were not differentially regulated and were similarly induced in BMdMs issued from either Balb/c or C57Bl/6 (**Figure 4**). Additionally, the transcription of HO-1, which can drive the phenotypic shift to M2 macrophages (Naito et al., 2014) and favor parasite survival (Pham et al., 2005; Luz et al., 2012), peaked earlier, lasted longer, and was not repressed by the parasites in BMdMs from resistant mice. However, HO-1 has, in addition, significant anti-inflammatory effects (Vijayan et al., 2018; Costa et al., 2020) and induces protective enzymes sequestering iron ions (Loboda et al., 2016).

Besides HO-1, GSH systems, one of the two major thiol-dependent antioxidant mechanisms in cells, are also activated by *L. major* infection. Our results also point out that initial contact with *Leishmania* was necessary and sufficient to promote the macrophage activation of the Nrf2 pathway. This pathway is therefore likely triggered by the stimulation of the receptors implicated in the recognition of the parasites, as heat-inactivated parasites also have the ability to induce the transcription of both NRF2 and its target genes.

All of the selected target genes were induced in both Balb/c and C57Bl6 BMdMs. However, the transcriptions of the cysteine transporter (Slc7a11) and of Gsr were differentially regulated between the macrophages from the two mouse strains. Indeed, these genes were more highly transcribed in C57Bl6 BMdMs. This increased transcription of Slc7a11, which is required to maintain the intracellular level of GSH and that of Gsr, involved in the recycling of oxidized glutathione, may result in glutathione accumulation, which has been reported to play a role in the resistance to *Leishmania* infection. Indeed, the depletion of glutathione has been shown to significantly increase the parasite load in the footpads of C57BL/6 mice infected with *L. major* (Cruz et al., 2008). Moreover, a close correlation was observed between the levels of intracellular soluble GSH and the secretion of nitrite (Buchmüller-Rouiller et al., 1995). In fact, a reduced cellular environment is important for several cellular functions, such as immune response and macrophage polarization (Fraternale et al., 2017). Thus, in contrast to HO-1, the transcription of Slc7a11 and Gsr, belonging to the glutathione system, was differentially regulated in BMdMs from resistant and susceptible mice, suggesting that glutathione may play an important role in the elimination of the parasite.

The second most striking observation is that, although the transcription of all the targeted antioxidant genes was induced by the infection and the triggering of the receptors, it was actively repressed by the parasite. This was particularly true for CD36 and CAT, a peroxide-scavenging enzyme, and for Gsr in BMdMs from susceptible mice.

CD36 represents an important part of the innate immune defense and may act as a pattern recognition receptor, in particular against bacterial pathogens (Areschoug and Gordon, 2009). CD36 is also important for host resistance to infection, and its deficiency significantly reduces mycobacteria burden (Hawkes et al., 2010), whereas its induction helps control severe malaria through parasite clearance (Olagnier et al., 2011). Increased transcription of CD36 has been reported for *Leishmania infantum* (Gatto et al., 2020) and *Leishmania amazonensis* (Okuda et al., 2016). However, while CD36^−/−^ macrophages are infected but do not support *L. amazonensis* proliferation, *L. major* amastigotes do not recruit CD36 and proliferate normally in CD36^−/−^ macrophages (Okuda et al., 2016). This is consistent with our finding that, despite their ability to induce NRF2 expression, *L. major* promastigotes actively limited CD36 transcription. In a different model, CD36 has been implicated in the regulation of ROS. Indeed, in murine vascular smooth muscle cells (VSMCs), while ROS induced the activation of the NRF2 target genes to limit oxidant stress, it also led to the generation of specific CD36 ligands, such as MP and oxLDL, allowing the scavenger receptor to phosphorylate NRF2 and, thus, to induce its exit from the nucleus and degradation (Li et al., 2010).

Thus, except for HO-1, the transcription of all the Nrf2 target genes tested was repressed, if not at all time points at least at the later ones, suggesting that these enzymes may play a role in limiting parasite survival and/or growth.

Our result showing that the silencing of the Nrf2 TF induced an increase in the parasite load is therefore in agreement with the parasite repressing the expressions of NRF2 target genes.

The role of NRF2 has also been explored, and transcriptomic and proteomic analyses highlighted the importance of Nrf2 signaling in cutaneous leishmaniasis (Vivarini et al., 2017; de Menezes et al., 2019). Indeed, the activation of NRF2 induced by *L. amazonensis* and *Leishmania braziliensis* enhanced the intracellular pathogen survival and disease progression (Vivarini et al., 2017). *L. amazonensis*-infected bone marrow macrophages exhibited increased expressions of NRF2 and antioxidant HO-1, which, together with higher levels of holotransferrin (holoTf) in parasitophorous vacuoles, contributed to the persistence of *L. amazonensis* infection (de Menezes et al., 2019). In our study, however, the silencing of NRF2 induced the persistence and multiplication of *L. major* parasites. This is in accordance with earlier results showing that treatment of *L. major*-infected BALB/c mice with *N*-acetyl-l-cysteine (NAC), a glutathione precursor and an NRF2 inducer, reduced the parasitism in their footpads (Rocha-Vieira et al., 2003).

We show in the present study that, while the master regulator of antioxidant response genes, Nrf2, is strongly induced by macrophage–*Leishmania* contact, the parasite represses the transcription of antioxidant genes and that the inactivation of the Nrf2 TF favors parasite survival and multiplication. Moreover, NRF2 target genes appear to play contrasting roles in *Leishmania*-infected macrophages, with HO-1 enabling parasite survival and enzymes of the GSH system, which are differentially regulated between BMdMs from resistant and susceptible mice, promoting parasite removal.

## Data Availability Statement

The original contributions presented in the study are included in the article/supplementary material. Further inquiries can be directed to the corresponding author.

## Ethics Statement

The animal study was reviewed and approved by the Ethics Committee of Institute Pasteur of Tunis, with ethics approval no. 1204.

## Author Contributions

HB, SR, CH, and CB carried out the experiments. MB helped with the flow cytometry experiments. DP and BT performed the qRT-PCR. IR supervised and contributed to the interpretation of the results. LG-T conceived and planned the experiments and wrote the manuscript. All authors contributed to the article and approved the submitted version.

## Funding

This study was funded by the Tunisian Ministry of Higher Education.

## Conflict of Interest

The authors declare that the research was conducted in the absence of any commercial or financial relationships that could be construed as a potential conflict of interest.

## Publisher’s Note

All claims expressed in this article are solely those of the authors and do not necessarily represent those of their affiliated organizations, or those of the publisher, the editors and the reviewers. Any product that may be evaluated in this article, or claim that may be made by its manufacturer, is not guaranteed or endorsed by the publisher.
